# How Serotonin Level Fluctuation Affects the Effectiveness of Treatment in Irritable Bowel Syndrome

**DOI:** 10.7759/cureus.9871

**Published:** 2020-08-19

**Authors:** Ilmaben S Vahora, Nicholas Tsouklidis, Rajat Kumar, Ravi Soni, Safeera Khan

**Affiliations:** 1 Internal Medicine, California Institute of Behavioral Neurosciences & Psychology, Fairfield, USA; 2 Medicine, California Institute of Behavioral Neurosciences & Psychology, Fairfield, USA; 3 Health Care Administration, University of Cincinnati Health, Cincinnati, USA; 4 Medicine, Atlantic University School of Medicine, Gros Islet, LCA; 5 Ophthalmology, California Institute of Behavioral Neurosciences & Psychology, Fairfield, USA; 6 Neurology, California Institute of Behavioral Neurosciences & Psychology, Fairfield, USA

**Keywords:** ibs, serotonin

## Abstract

Irritable bowel syndrome (IBS) is one of the most commonly diagnosed functional gastrointestinal (GI) disorders. It affects both men and women. Enteric serotonin (5HT) is responsible for gut motility, secretion, visceral hypersensitivity, and inflammation. The serotonin reuptake transporter (SERT) maintains serotonin levels by regulating its reuptake. An increase in SERT expression causes a decrease in serotonin, which leads to IBS-C (irritable bowel syndrome, constipation-predominant), whereas a decrease in SERT transcription causes an increase in serotonin, which leads to IBS-D (irritable bowel syndrome, diarrhea-predominant). Some factors can alter SERT transcription, such as certain bacteria, inflammation, growth factor, and glucagon-like peptide-1. This shows that 5HT and SERT both have an important role in IBS pathophysiology so that it would be a better subject to target for the treatment aspect of IBS. 5HT3 receptor antagonists are advisable for IBS-D to block the excessive activity of serotonin at the 5HT3 receptor and reduce gut motility. For IBS-C, we prescribe 5HT4 receptor agonists, which promote gut motility. Also, the latest treatment approach, antidepressant drugs TCAs (tricyclic antidepressants) and SSRIs (selective serotonin reuptake inhibitors), are helpful by modulating serotonin levels in the gut. In this literature review, we found that serotonin is one of the main pathophysiological factors for IBS, and we can treat IBS by targeting serotonin function on gut motility.

## Introduction and background

In the United States, almost 10 to 15% of the adult population suffers from irritable bowel syndrome (IBS) symptoms, and out of those, adults that have been diagnosed with the disease are only 5 to 7%. IBS is one of the most commonly diagnosed disorders in gastroenterology, as well as in primary care practices [1]. Around 12% of patient visits in primary care are for IBS [2] and 20 to 50% of gastroenterologist patient visits [3]. IBS is 1.5-2 times more commonly seen in women than in men and is most commonly diagnosed in people under the age of 50 [1]. 

IBS is a chronic, recurring, and remitting functional disorder of the gastrointestinal (GI) tract characterized by abdominal pain, distention, and changes in bowel habits with no structural abnormalities [4]. According to Rome IV criteria, IBS diagnosed with recurrent abdominal pain is associated with two or more of the following criteria: (a) related to defecation, (b) change in frequency of stool, and (c) change in consistency of stool, at least one day per week in the last three months. Criteria must be fulfilled for the last three months, with symptom onset at least six months before diagnosis [5]. According to the predominance of symptoms presentation such as stool frequency and consistency, IBS is divided into four types: IBS with constipation (IBS-C), IBS with diarrhea (IBS-D), IBS mixed (IBS-M), and IBS unclassified [5]. There is no single diagnostic test for IBS, with diagnosis mainly being based on symptom presentation. Currently, research is being conducted for the effectiveness of antidepressants for IBS symptom improvement. Researchers found that about 55% of patients treated with selective serotonin reuptake inhibitors (SSRIs) showed improvement in IBS symptoms vs. 33% for placebo [6].

The exact pathophysiology and treatment options of IBS are still not known [7]. Pathophysiology of IBS is multifactorial and includes abnormality in GI motility, serotonin reuptake transporter (SERT) gene transcription, alteration of the human microbiota, stress, brain-gut interaction, visceral hypersensitivity, and low-grade inflammation [8,9,10]. Many options are available for treatment, like dietary changes such as the FODMAP diet, low fermentable disaccharides, oligosaccharides, monosaccharides, psyllium, and wheat bran and psychological therapies such as cognitive behavior therapy and hypnotherapy. Pharmacological options such as laxatives, antispasmodics, serotonergic agents, antidepressants, loperamide, rifaximin, and cholestyramine are available, and herbal therapies, acupuncture, and traditional Chinese medicine (TCM) have also been used. Patients with IBS are treated based on the predominance of their symptoms. However, an exact treatment plan is not known [11]. Since the cause of IBS is not exactly identified, it is difficult to screen for the disease, and on the same point, it is hard to look for preventive measures [12]. There is still less data available on stool specific subcategories in IBS patients [7].

In this review article, we will mainly focus on how an abnormality in SERT transcription alters serotonin levels in the gut lumen [10], which changes gut motility and function. We will also see treatment options, mainly targeting 5HT receptors.

## Review

Methods

We systematically searched online databases such as PubMed, Google Scholar, PubMed Central (PMC), and MEDLINE. We searched for all kinds of studies showing an association of irritable bowel syndrome and serotonin. We used IBS, serotonin, SERT, 5HT3 receptor, diarrhea, constipation, abdominal pain, 5HT3 antagonist, 5HT4 agonist, management of IBS, and antidepressant as keywords alone and in combination to look for published papers from the last five years in the English language. First, we scanned articles through the title and found 100 articles from 200 articles. Then, by reading the abstracts and removing duplicate papers, we ended up with 50 studies. After that, we applied the inclusion and exclusion criteria, which gave us 30 papers. After reading the full-text articles, we ended up with 26 studies for the final review.

Inclusion and Exclusion Criteria

The papers are sourced from the last five years and published in the English language. Papers only showing the relationship between serotonin and IBS were included. All types of studies, both male and female gender, all kinds of population and geographical area, and only full-text articles were included. There were not any age criteria included. Unpublished studies or research papers in other languages were excluded. A few articles were added from within the references of selected studies that were not from the past five years.

Result

Twenty-six studies were finalized for the review article [13-38]. Ten of them were review articles [13,14,16,25-27,29,33,36,38], with two systematic reviews on ramosetron and 5HT3 antagonist efficacy on IBS, respectively [27,29]. Review articles included studies that were published more than five years ago. One of them was an animal study showing the relation between LGG and post-infectious IBS (PI-IBS) [19]. There were five randomized controlled trials (RCTs) [28,30,32,35,37]. Two RCTs evaluated the role of ondansetron in IBS [32,35]. One RCT compared alosetron vs traditional pharmacotherapy in IBS [30]. The remaining two RCTs showed the effect of ramosetron and antidepressants for symptoms of IBS [28,37]. There were four in vitro trials included in the review [15,18,23,24]. One of those in vitro trials showed how NK2/NK3 receptors control serotonin release [15]. One of the in vitro trials discussed the serotonin reuptake transporter (SERT) and IBS relationship [18], and the remaining two in vitro trials displayed how tumor growth factor-beta one and glucagon-like peptide-1 modulate SERT transcription, respectively [23,24]. One was an observational study, a case-control trial of ramosetron in IBS patients that showed improvement in IBS symptoms, fecal incontinence, and urgency [31]. 

Discussion

Pathophysiology of irritable bowel syndrome is undetermined [7], but serotonin plays a major role in gut motility and its pathophysiology. Serotonin is one of the primary neurotransmitters for maintaining gut distension, motility, and visceral sensitivity. We will discuss here how serotonin forms and its role on gut motility, the factors that can fluctuate the level of serotonin in the gut, how SERT maintains serotonin level in the gut, which factors affect SERT transcription, and lastly treatment of IBS targeted on 5HT receptors and serotonin reuptake mechanism. Our target in this discussion section is to shed some light on “How serotonin level fluctuation affects the effectiveness of treatment in irritable bowel syndrome.”

Role of Serotonin on Gut Motility

The study conducted by Terry et al. showed that 95% of serotonin formed in the intestine is for endocrine, autocrine, paracrine, and hormonal functions. 5HT (serotonin) regulates enteric nervous system (ENS) neurogenesis and development, inflammation, secretion, sensation, motility, and epithelial development [13].

**Figure 1:**
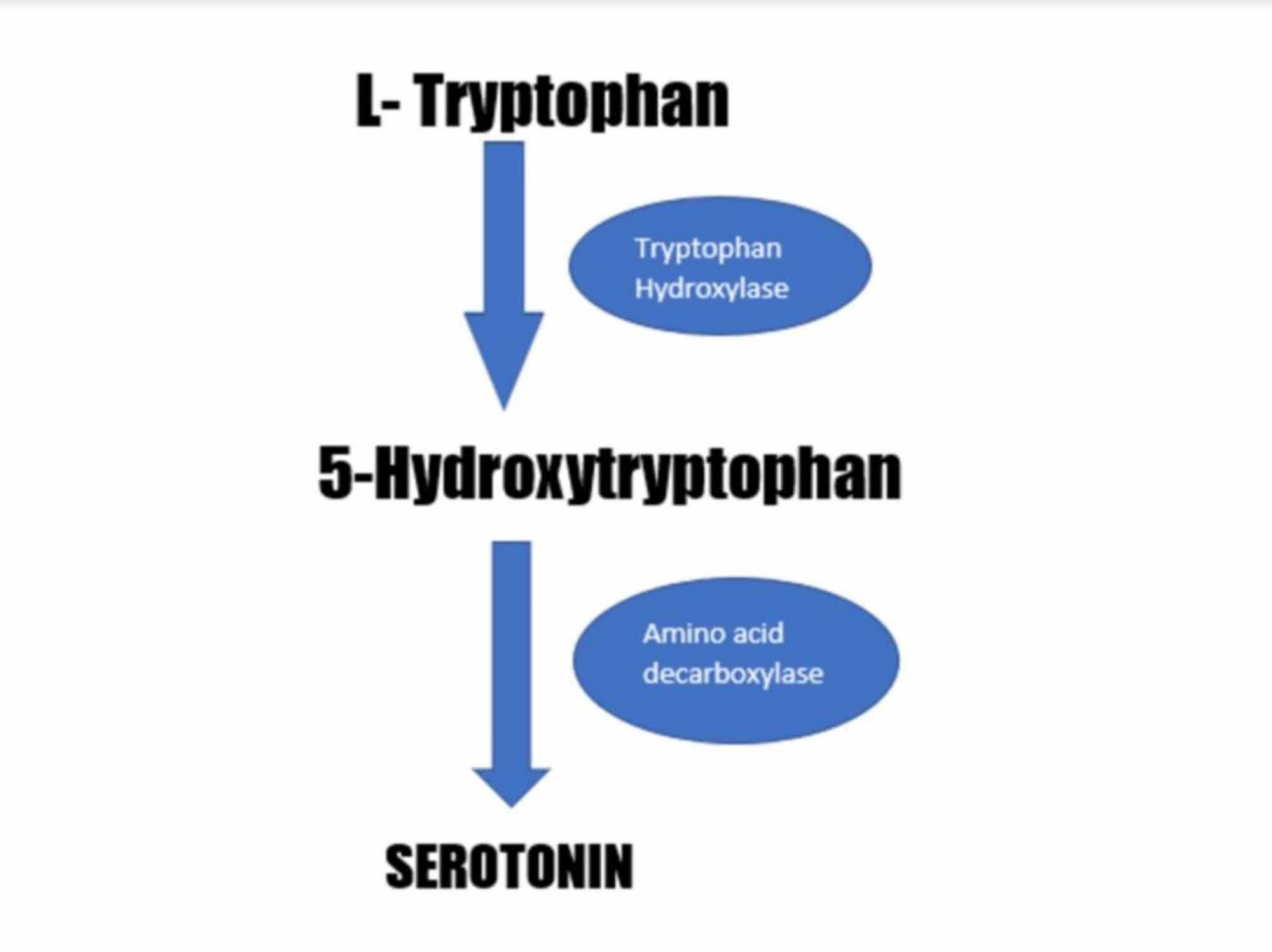
Serotonin formation with the help of tryptophan hydroxylase and amino acid decarboxylase enzymes

Serotonin synthesis starts with L-tryptophan, which is converted into 5-hydroxytryptophan (5-HTP) by the enzyme tryptophan hydroxylase (TPH). 5-HTP is converted into 5HT by aromatic amino acid decarboxylase. There are two types of TPH: TPH1 and TPH2. TPH1 produces serotonin in the intestinal enterochromaffin (EC) cells and is responsible for 90% of intestinal serotonin production. The remaining 10% of serotonin production is by TPH2, which is located in neurons of the enteric nervous system and central nervous system. Figure 1 represents serotonin formation with the help of enzymes. 

Apart from 15 types of 5HT receptors, 5HT3 and 5HT4 receptors are studied the most. 5HT3 is present on the sensory and myenteric neurons and 5HT4 is present on presynaptic neurons. The word 'serotonin’ itself indicates its meaning, to increase tone or cause contraction. They also mentioned in their study that serotonin increases peristalsis, motility, and propulsive contraction by acting on the 5HT3 receptor and decrease motility by acting on the 5HT4 receptor [13]. The study by Padhy et al. argued afferent and efferent neurons of the myenteric plexus present in layers of intestinal wall secrete serotonin, which causes an increase in firing rate of the secretomotor neurons and an increase in intestinal motility and secretion. Patients with irritable bowel syndrome - diarrhea subtype have increased serotonin production and availability in the gut, which increases motility and causes diarrhea and pain. In the same study, they proposed that serotonin levels are increased more in the gut in constipation dominant IBS compared to diarrhea dominant IBS [14].

Enck et al. showed the role of microbiota and serotonin; intestinal bacteria are required for the synthesis of serotonin [12]. The microbiota needed for serotonin synthesis is the clostridial class from the Firmicutes phylum [12]. Kojima et al. have discussed the effect of neurokinin 2 and neurokinin 3 (NK2/NK3) on serotonin release. The NK2 agonist tachykinin works on the NK2 receptor in guinea pig colon and enhances serotonin secretion by their agonist action at the receptor. Tachykinin NK3 receptor agonists also raise the 5HT level by firing on the NK3 receptor, but there is no effect of the NK1 receptor on serotonin levels. The NK3 receptor is located on myenteric and submucosal plexus neurons and the NK3 receptor plays a crucial role in inflammation. Serotonin levels in the colon were 10 times higher in IBS than control, thus serotonin causes symptoms of IBS such as abdominal pain, nausea, and vomiting. Kojima et al. analyzed the role of the NK2/NK3 receptor on serotonin release from EC cells and the development of IBS symptoms. The role of tachykinin on the NK2/NK3 receptor cascade causes increase serotonin-induced gut motility in IBS patients via acting on serotonin receptor subtypes [15]. 

Kending et al. mentioned how serotonin altered colonic motility in their study [16]. They discussed certain important factors such as serotonin release by EC cells through mechanical and chemical stimuli, agonist activity at 5HT3 and 5HT4 receptor increases colonic peristalsis and propulsion, and antagonist activity at 5HT3 and 5HT4 receptor decreases colonic peristalsis. Based on that, we can conclude that serotonin is able to generate colonic peristalsis [16]. Heredia et al. proposed TPH1-/- knockout mice have decreased serotonin levels in the intestine; thus, fecal pellets from these mice were larger than normal pellets because of lack of serotonin-induced peristalsis [17]. The exact mechanism of how serotonin increases or decreases gut motility is not known. Future studies are required to discuss this aspect in detail. Certain studies show that 5HT3 increases motility and 5HT4 decreases motility, although the exact action of the receptors has not been studied.

SERT Transcription and IBS

**Figure 2:**
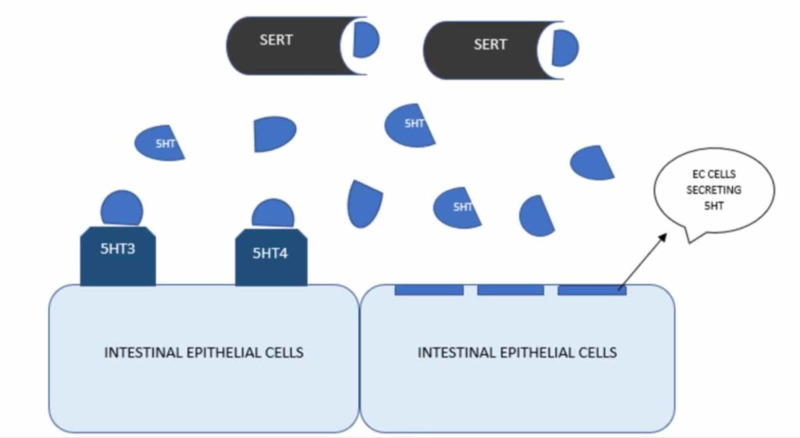
Mechanism showing how SERT maintains serotonin levels by its reuptake. 5HT - Serotonin, SERT - Serotonin Reuptake Transporter, EC cells - Enterochromaffin cells

The traditional review article conducted by Jin et al. described that after release from EC cells, serotonin deactivation occurs by serotonin reuptake transporter (SERT) to maintain the appropriate level of serotonin in the gut. Figure 2 shows the mechanism of action of SERT for serotonin reuptake. SERT takes serotonin into enterocytes, where it is catabolized by monoamine oxidases. So, any changes in SERT transcription will alter available serotonin in the gut. In patients with IBS, there is a decreased transcription of SERT resulting in elevated serotonin level, which ultimately causes diarrhea and discomfort, which is transmitted by serotonin through the gut-brain axis. Both genetic and environmental factors alter SERT transcription, but environmental factors affect SERT transcription more than genetic factors [18]. 

Cao et al. showed serotonin signaling and the role of SERT in the reuptake of serotonin in their study. SERT has two types of polymorphisms. The first one is the 5HT transporter gene-linked polymorphic region (5-HTTLPR), and the second one is STin2. 5-HTTLPR is located on SLC6A4; chromosome 17q11.1-q.12, and it is divided into long alleles (L) and short alleles (S). The transcription efficiency of the L/L genotype is much higher than that of the S/S and L/S genotypes. There is elevated SERT expression seen in IBS-C compared to IBS-D. The L/L genotype is more commonly seen in IBS-C than IBS-D, whereas the S/S genotype is present commonly in IBS-D. The expression of SERT is elevated in IBS-C and lower in IBS-D, thus causing a decrease and increase in the serotonin level in the gut, respectively [19]. Contradictory to that, studies conducted by Sikander et al. and Pata et al. showed that the S/S genotype is associated with IBS-C patients in India and the Turkish population [20,21]. Jia et al. mentioned that there is no association between 5-HTTLPR and IBS in the Chinese population [22]. The second SERT gene polymorphism is called a variable number of tandem repeats STin2 (STin2 VNTR). 10/12 genotypes of STin2 might be related to IBS [18].

Factors that can alter SERT transcription are *Lactobacillus rhamnosus* bacteria (LGG-s), glucagon-like peptide, tumor growth factor-beta 1 (TGF-B1), immunity, inflammation, growth factors, and microbiota. These factors might modify the bioavailability of serotonin through changing SERT transcription. The animal study conducted by Cao et al. showed mice infected with *Campylobacter jejuni* developed post-infectious IBS (PI-IBS). Mice infected with *C. jejuni* were found to have decreased SERT mRNA expression 0.7-fold compared to the control group. The group of mice that received treatment with LGG-s was divided into different groups based on doses. Groups received undiluted LGG-s, double diluted LGG-s, and triple diluted LGG-s. The observed outcome showed that the rats receiving more diluted SERT transcription in the mice with the disease as compared to the ones receiving less diluted doses [19]. Jin et al. argued that in PI-IBS and IBS-D, there is hyperplasia of EC cells and reduced SERT activity and increased serotonin level. Infection with *Trichinella spiralis* also decreases SERT transcription [18]. An in vitro study conducted by Nazir et al. showed TGF-B1 increases SERT activity via phosphatidylinositol 3-kinase (P13K), and compared to control TGF-B1 also upgrades SERT expression by more than 2-fold. In the same study, they have mentioned, via altered membrane trafficking events such as exocytosis or endocytosis, TGF-B1 increases SERT function and surface levels [23]. It has also been found to effect growth factors and gut microbiota on SERT transcription. Jin et al. proposed the epidermal growth factor (EGF) binding to the epidermal growth factor receptor activates the hSERT (human serotonin transporter) promoter and increases SERT mRNA transcription. They discovered that there is a decrease in EGF levels in IBS patients that leads to lower SERT transcription and increased 5HT levels in the gut, producing visceral hypersensitivity and IBS. They also discussed microbiota relation to SERT. In the fecal sample of IBS-D, there is a decrease amount of these microbiotas such as bifidobacterium, lactobacillus, actinobacteria, and lactobacillus, whereas microbiota such as proteobacteria and firmicutes are increased in a fecal sample. Microbiota affects tryptophan metabolism and altered the 5HT level in the gut [18]. The in vitro study conducted by Cui et al. argued glucagon-like peptide 1 (GLP-1) analog exendin-4 modulates 5HT level in intestines by increasing SERT transcription, decrease visceral hypersensitivity, and abnormal gastrointestinal motility. SERT expression is reduced in an IBS patient’s rectum and colon, so the 5HT level is increased in the gut, especially in IBS-C. Exendin, which is a 54% analog of GLP-1 with a better half-life, improves SERT expression in IBS and controls 5HT levels. In this study, they measured SERT transcription by reverse transcription-quantitative polymerase chain reaction (PCR) and western blotting, after giving 10 and 100 nM doses of exendin. The result showed an increase in SERT transcription in intestinal epithelial cells, activity, and the rise of 5HT uptake. GLP-1 analog binds to the GLP-1 receptor to exert its action. If cells are pretreated with GLP-1R antagonist, there is no rate change in SERT transcription [24].

By discussing the importance of SERT transcription related to IBS, we can say that treatment targeting SERT can help treat IBS symptoms, and future research necessitates study of this aspect. Several more questions need to be studied further, such as how GLP-1 stimulates SERT transcription, and as some studies have shown an increase in 5HT in IBS-C in contrast to others that have shown an increase in 5HT in IBS-D, these aspects also require future discussion. 

Treatment of IBS Targeting 5HT Receptor

As we discussed earlier, serotonin has many receptor subtypes, but 5HT3 and 5HT4 receptors are most commonly discussed. Treatment options for IBS focusing on these receptors will be discussed under this subheading. 5HT4 agonists are mostly used for IBS-C, and 5HT3 antagonists are treatment options for IBS-D.

5HT4 Agonist Agents

*Prucalopride*: Sinagra et al. discussed prucalopride; it works as a 5HT4 agonist, enhances colonic motility and improved colonic stool frequency and consistency in a patient with chronic constipation. They argued based on different RCT that prucalopride increases complete spontaneous bowel movement (CSBM) per week, alleviates disease severity, and improves the quality of life in chronic constipation patients. Prucalopride is not arrhythmogenic. Even at high doses, prucalopride does not cause any cardiovascular adverse effects, such as QT prolongation, and this also applies to elderly patients [25]. This drug has minor side effects such as headache, nausea, diarrhea, and abdominal pain. Worsening abdominal pain restricts its use in clinical practice [25].

*Renzapride:* Renzapride is a full agonist at 5HT4 and an antagonist to 5HT2B and 5HT3 receptors. Renzapride showed no efficacy in the improvement of bowel emptying and IBS symptoms compared to placebo. This drug causes more diarrhea and ischemic colitis as adverse drug reaction (ADR) as compared to the placebo. There are no cardiac side effects with this drug [25].

*Velusetrag: *The study conducted by Sinagra et al. showed that velusetrag is an effective option for IBS-C treatment. It is a 5HT4 agonist and its binding affinity for 5HT4 receptors is 500-fold more than other 5HT4 agonist agents. The mechanism of action of this drug is to increase contractility of the antrum, fundus, duodenum, and jejunum. Velusetrag was given to chronic constipation patients at different doses for different durations; they noticed increase colonic motility, stool frequency, and consistency. There was also an improvement in CSBM and in symptoms of chronic idiopathic constipation. No significant adverse drug effects were noticed except diarrhea, headache, and nausea [25].

*Tegaserod:* A 5HT4 receptor agonist, tegaserod is efficacious for IBS symptoms. It alleviates abdominal pain, bloating, discomfort, and constipation. It also improves the quality of life and work productivity. It is currently prescribed in the USA. Diarrhea, cramping, and rare cardiovascular (CVS) side effects are seen with this drug [26].

5HT3 Antagonist Agents

5HT3 is present on intestinal plexuses, sympathetic and parasympathetic, and on sensory nerves to stimulate the release of neurotransmitters. 5HT receptors bind to 5HT3 receptors on parasympathetic ganglia, cause intestinal contraction and secretion by stimulating nerve terminal acetylcholine release. Activation of the 5HT3 receptor causes contraction. 5HT3 antagonists inhibit its activation, decreasing depolarization of the extrinsic sensory neuron and its signal transferring to the brain, alleviate abdominal pain and discomfort [27].

*Ramosetron:* Ramosetron is a 5HT3 antagonist. The 5HT3 receptor increases colonic motility and causes diarrhea, nausea, and vomiting. It is beneficial to treat IBS-D symptoms in both men and women by blocking serotonin activity at the 5HT3 receptor. After giving ramosetron to 296 men with IBS-D, it was shown that there is an improvement in bowel habits, abdominal pain/discomfort, urgency, and stool frequency [25]. Fukudo et al. showed the efficacy of ramosetron in the treatment of D-IBS in women in their RCT. Ramosetron and alosetron have been approved to treat D-IBS only in men. However, in this study, they demonstrated the effectiveness of ramosetron in women. They selected a sample size of 580 women amongst 296 were patients who took 2.5 mcg of ramosetron daily for 12 weeks and they looked for an improvement in overall symptoms of IBS and stool consistency as the primary endpoint. As the secondary endpoint they looked for relief of abdominal pain/discomfort, stool frequency, urgency and feeling of incomplete evacuation and improvement in IBS-QOL. They found that for both primary endpoints, ramosetron worked excellent over placebo. All targeted secondary endpoint symptoms are relieved better with ramosetron compare to placebo [28]. A systematic review based on RCT of ramosetron conducted by Qi et al. showed ramosetron is equally efficacious for both men and women [29]. Patients treated with ramosetron were found to have constipation and hard stool more often compared to placebo, whereas some patients had anemia and infectious enterocolitis as rare side effects [28]. The incidence of constipation was much less in the ramosetron-treated group than a patient treated with alosetron and cilansetron [27].

*Alosetron:* A randomized controlled trial study conducted by Olden et al. discussed the effectiveness of alosetron in IBS-D patients' symptoms and quality of life. Alosetron is a 5HT3 antagonist and decreases colonic motility by blocking serotonin activity on 5HT 3 receptors. In this study, 1956 patients with severe IBS-D were selected and randomized to the treatment group of 1 mg alosetron twice daily or control. Work productivity and resource were measured by filling a standard questionnaire, health-related quality of life (HRQOL), IBS quality of life (IBSQOL), and IBS symptoms by global improvement scale (GIS). The patient treated with alosetron had fewer clinic visits, decreased use of over the counter medicines to treat IBS, and less decrease in their loss of productive workdays [30]. They also showed decreased social isolation, decreased restriction on their outdoor activities, and overall global improvement, as compared to the patients taking only placebo [30]. The incidence of adverse events was not significant and were comparable to the ones previously reported [30]. Lacy et al. conducted a prospective, open-label, multicenter observational study to evaluate alosetron use in severe IBS-D patients who have not improved adequately to other treatments. They used the FDA composite stool consistency parameter for end results. Patients who met criteria for severe IBS-D, which were 192 from 256, were given alosetron 0.5 mcg twice daily for 12 weeks. At the end of the study, they found an overall treatment responder rate of 44.6% with high-level control of abdominal pain, fecal urgency, diarrhea, and incontinence. At the end of the study, 39.6% of the patients did not report fecal urgency [31]. Side effects of alosetron are hard stool, constipation, and also some serious side effects of ischemic colitis [30]. Whereas Lucy et al. mentioned no serious adverse effects such as colonic ischemia, constipation, or death that occurred during their clinical trial [31]. Zheng et al. showed in their meta-analysis on randomized controlled trials study, alosetron had nine cases of ischemic colitis than the control group. Because of that alosetron is restricted to use in patients with severe refractory IBS-D [27].

*Ondansetron:* Gunn et al. conducted a randomized controlled trial, cross-over trial of five weeks of ondansetron and placebo in 125 patients diagnosed with IBS-D and 21 controls. The levels of 5-HIAA, 5HT, and 5HIAA/5HT were measured in IBS-D patients and control before starting treatment, and it was higher in IBS-D patients than control [32]. The super responder was who had the greatest sensitivity to ondansetron with <4 mg dose. That was low in the group requiring <4 mg dose compared to those who received >4 mg and controls, and this could be because of decreasing synthesis or increasing turnover. Also, in stool from the responder, they found the homozygous CC genotype was present in 33% of the responders [32]. Ondansetron improved fecal urgency (p<0.001), bloating (p=0.002), and frequency (p=0.001), but had no effect on abdominal pain [33]. In contrast to a study conducted by Goldberg et al. in 1996, showed ondansetron relieved pain perception by increasing rectal sensory threshold to electrical stimulation, pain episodes were decreased after taking ondansetron [34]. Constipation is the most common side effect of ondansetron, which can be overcome by dose titration. It has no incidence of ischemic colitis as do other 5HT3 antagonists [35]. Also, headache, backache, abdominal pain, and rectal bleeding side effects were seen [33].

*Serotonin modulators:* Selective serotonin reuptake inhibitors, serotonin-norepinephrine reuptake inhibitors, and tricyclic antidepressants (TCAs) all decrease serotonin reuptake and increase its availability by modulating its levels.

SSRIs and TCAs change levels of serotonin in the GI tract and improve IBS symptoms and abdominal pain. Both change central nervous system (CNS) response to pain perception and might reduce activation of pain center and reduce pain processing. TCAs increase oro-cecal and intestinal transient time, so are used in IBS-D. SSRIs decrease oro-cecal and intestinal transient time and are used in IBS-C. Amitryptiline improved incomplete defecation and loose stools compared with placebo, but because of ADR such as drowsiness, dry mouth, and greater cost of SSRI, it is not prescribed [36,26]. Camilleri mentioned in his review article on duloxetine RCT that it improves IBS symptoms, abdominal pain, bowel dysfunction, and quality of life [36]. Xie at al. proposed in their meta-analysis that SSRIs are not superior to placebo to relieve IBS global symptoms. TCAs prove to be beneficial in alleviating IBS symptoms such as abdominal pain, discomfort, bloating, and change in bowel habit.

They also mentioned because of side effects like dry mouth, palpitation, and drowsiness of TCAs and poor sleep, anxiety, headache, and nausea of SSRIs, these drugs are not advisable to IBS patients [37]. Chen et al. argued in their study that SSRIs showed moderate improvement in IBS symptoms and should be advisable in patients with comorbid depression [38]. It is still controversial whether SSRIs are actually useful for the treatment of IBS or not. Agitation, nausea, and insomnia are concerning ADR of SSRIs [38].

Serotonergic agents have been found to be effective in improving IBS, but it is not completely curable. Future evaluation is required to find drugs that can cure this disease. Although these drugs alleviate symptoms, their efficacy is not known. Further studies are required to know if antidepressants cause a change in symptoms because of an improvement of psychological symptoms or improvement in IBS symptoms themselves. Studies on the serotonergic agent efficacy on IBS-M are also required in the future. Some of the studies that discussed the role of serotonin and SERT in IBS and the medicines used in IBS are summarized in Table 1. 

**Table 1:** Some of the studies included in the review RCT - Randomized controlled trial, TCA - Tricyclic antidepressants, IBS-D - Irritable bowel syndrome diarrhea-predominant, 5HT - serotonin, TPH - Tryptophan hydroxylase, SERT- Serotonin reuptake transporter, TP - Traditional pharmacotherapy, HRQOL - Health-related quality of life

Name of authors	Purpose of the study	Type of study	Result/conclusion
Terry et al. [13]	The study was conducted to find “5-HT in intestinal motility and inflammation as well as its function as a hormone in osteocyte homeostasis.”	Review article	Serotonin is an important modulator of intestinal motility and secretion.
Jin et al. [18]	The study conducted to show” current insights regarding the regulation of SERT in IBS, including aspects of SERT gene polymorphisms, microRNAs, immunity, and inflammation, gut microbiota, growth factors, among others.”	In vitro animal study	In vitro mice study concluded there is major Colonic motility dysfunction in mice with TPH.
Cao et al. [19]	Effects of lactobacillus rhamnosus GG supernatant on SERT in mice with PI-IBS	Animal study	LGGs upregulate SERT expression in the intestinal tissue of PI-IBS mice but not in brain tissue. It decreases the 5HT level, also an option to treat IBS.
Zheng et al. [27]	Efficacy and safety of 5HT3 antagonist on D-IBS	Systematic review and meta-analysis of RCT	There is an improvement in symptoms of D-IBS after treating with a 5HT3 antagonist.
Fukudo et al. [28]	Efficacy of ramosetron for D-IBS	RCT	Ramosetron improves symptoms of IBS and increases stool consistency and quality of life.
Olden et al. [30]	Alosetron VS traditional pharmacotherapy for D-IBS	RCT	Alosetron is better than TP for improvement IBS symptoms, HRQOL, participating in social and outdoor activities.
Gunn et al. [32]	Ondansetron effectiveness for treatment of D-IBS	RCT	They found “IBS‐D patients have significant abnormalities in mucosal 5‐HT metabolism. Those with the lowest concentration of 5‐HT in rectal biopsies showed the greatest responsiveness to ondansetron.”
Xie et al. [37]	Are Antidepressants beneficial for IBS-D?	RCT	TCAs showed to alleviate symptoms of IBS-D, whereas SSRIs showed no benefits.

Limitations

We faced a few limitations while conducting this traditional review. There were not enough randomized control trials found regarding the treatment aspect. There was less data found regarding 5HT4 agonist drugs, specifically the RCT trials. Not many articles were found showing a link between cisapride, mosapride, cilansetron, and IBS. Articles in languages other than English could not be included.

## Conclusions

We conducted this literature review to discover the role of serotonin in the pathophysiology of IBS, how serotonin levels fluctuate, and what drugs can fix these altered serotonin levels to alleviate IBS symptoms. We found that serotonin is one of the main factors to maintain gut motility, any changes in its level definitely modify gut motility, SERT is one of the factors that are responsible for altered serotonin level in the gut, and finally, serotonergic agents can change serotonin levels and maintain gut motility. In this paper, we tried to mention all the aspects of IBS related to serotonin, from its function on bowel movement to pathophysiology to the treatment perspective. This article would be beneficial to the research community to understand how serotonergic agents would still be one of the treatment options available for IBS. Still, there are a few questions that should further be investigated, as serotonin does alter gut motility, but the exact mechanism is yet to be discovered. SERT maintains serotonin level so as the gut motility, so any treatment perspective regarding SERT would be beneficial for IBS. Serotonergic agents are one of the treatment options for IBS, but their superiority over other options or between them is not proven and needs to be answered.
