# Correction: How Serotonin Level Fluctuation Affects the Effectiveness of Treatment in Irritable Bowel Syndrome

**DOI:** 10.7759/cureus.c36

**Published:** 2020-09-01

**Authors:** Ilmaben S Vahora, Nicholas Tsouklidis, Rajat Kumar, Ravi Soni, Safeera Khan

**Affiliations:** 1 Internal Medicine, California Institute of Behavioral Neurosciences & Psychology, Fairfield, USA; 2 Medicine, California Institute of Behavioral Neurosciences & Psychology, Fairfield, USA; 3 Health Care Administration, University of Cincinnati Health, Cincinnati, USA; 4 Medicine, Atlantic University School of Medicine, Gros Islet, LCA; 5 Ophthalmology, California Institute of Behavioral Neurosciences & Psychology, Fairfield, USA; 6 Nuclear Medicine, Vardhman Mahavir Medical College and Safdarjung Hospital, Delhi, IND; 7 Neurology, California Institute of Behavioral Neurosciences & Psychology, Fairfield, USA

This article originally contained the following statement in reference to ZELNORM (tegaserod): "It is not prescribed in the USA."

However, on August 15, 2019, ZELNORM (tegaserod) was reintroduced to the United States market. Therefore, the text has been amended to reflect that ZELNORM (tegaserod) is indeed currently prescribed in the USA. The authors and Cureus sincerely regret the error.

